# Supplementary material for: Concurrent disease burden from multiple infectious diseases and the influence of social determinants in the contiguous United States
Source: PLoS One. 2024 Sep 4;19(9):e0293431. doi: 10.1371/journal.pone.0293431 (PMC11373817; doi:10.1371/journal.pone.0293431)
Supplement: S1 File — (DOCX) [file pone.0293431.s001.docx]

**Supporting Information**

**S1 File**

**Map A. Central cluster counties with the highest relative risk.**

The map below shows the infectious disease that each county had the highest relative risk for. Central cluster counties are shown below with the highest prevalent disease, COVID-19, HIV, Influenza, or TB, even if it had a multiple infectious disease burden.


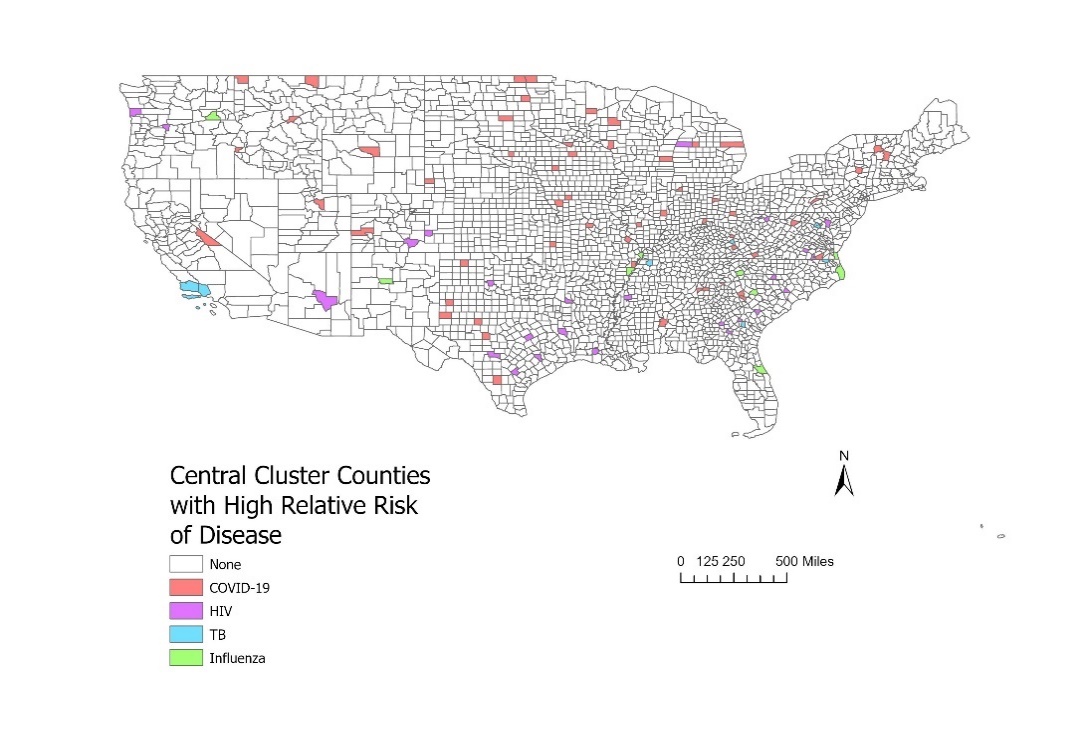


**Map B. Central cluster counties for multiple diseases.**

The map below shows the infectious disease that each county had the highest relative risk for and highlights counties with multiple infectious diseases as dark blue. This visual representation emphasizes multiple disease burden.

*
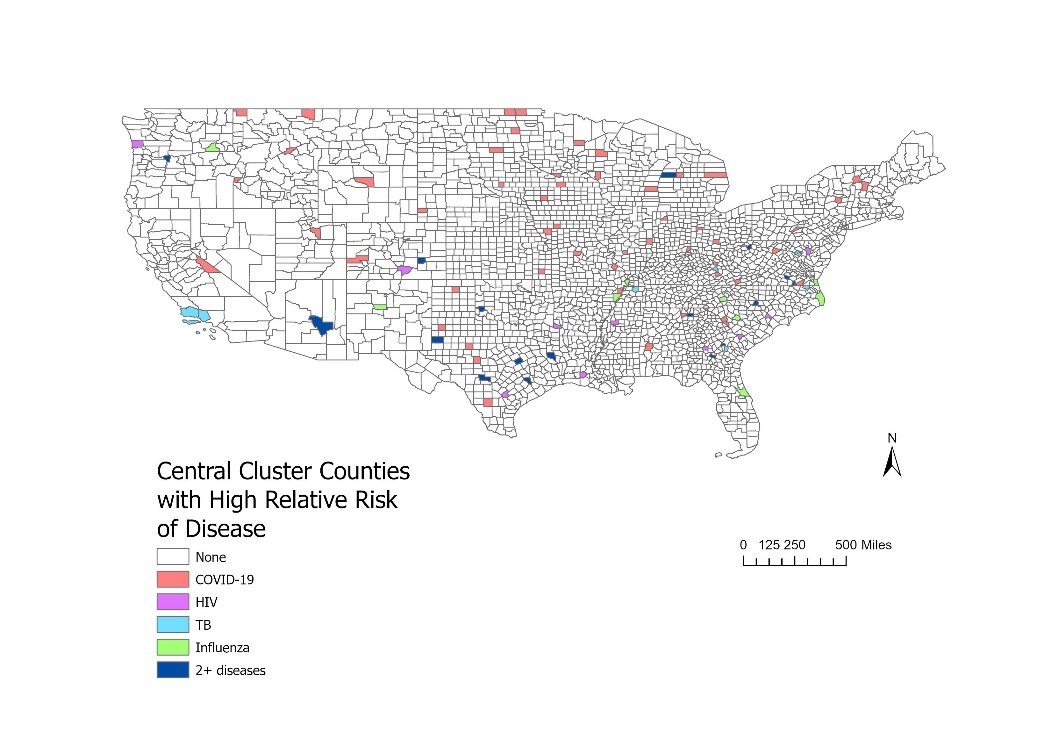
*

**Graph A. The number of high-risk clusters by percent of population below the poverty line.**

The graph below shows the number of counties by percentage of the population below the poverty line. This graph demonstrates the distribution of high-risk clusters among the population that is below the poverty line.

**
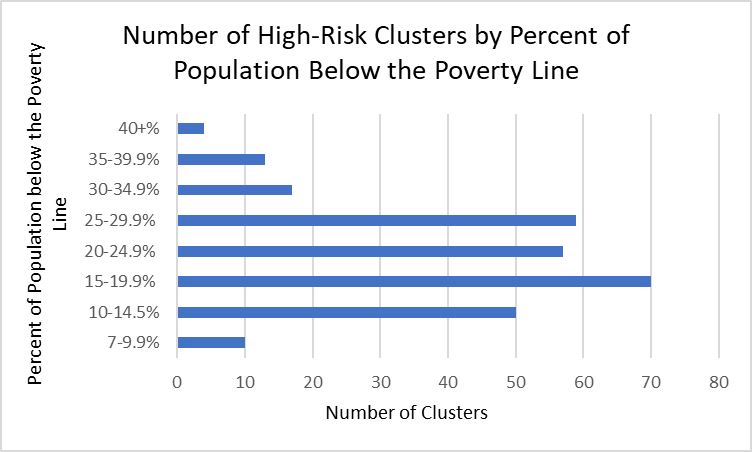
**
