# Supplementary material for: Concurrent disease burden from multiple infectious diseases and the influence of social determinants in the contiguous United States
Source: PLoS One. 2024 Sep 4;19(9):e0293431. doi: 10.1371/journal.pone.0293431 (PMC11373817; doi:10.1371/journal.pone.0293431)
Supplement: S2 File — (DOCX) [file pone.0293431.s002.docx]

**Supporting Information**

**S2 File**

**Table A. Number of high-risk counties with disease below US poverty level.**

The table below shows the number of counties found at the center of high-risk clusters by percent of that county which is 125% below US poverty level. This table corresponds to graph 1 of S1 File.

| ***Number of Hot Spot Counties with Disease by 125% Below US Poverty Level*** | | | | | | | | | |
| --- | --- | --- | --- | --- | --- | --- | --- | --- | --- |
|  | 7-9.9% | 10-14.5% | 15-19.9% | 20-24.9% | 25-29.9% | 30-34.9% | 35-39.9% | 40+% | Total |
| TB 2019 |  | 2 | 5 | 1 | 6 | 1 |  |  | 15 |
| TB 2020 |  | 3 | 4 | 2 | 5 | 1 | 1 |  | 16 |
| COVID-19 2022 | 5 | 19 | 20 | 13 | 18 | 3 | 1 | 1 | 80 |
| COVID-19 2021 | 3 | 23 | 22 | 22 | 7 | 5 | 4 | 3 | 89 |
| INFLUENZA 2021 |  |  | 1 | 1 |  |  |  |  | 2 |
| INFLUENZA 2020 |  |  | 1 | 1 |  |  |  |  | 2 |
| HIV 2019 | 1 | 1 | 8 | 8 | 11 | 3 | 4 |  | 36 |
| HIV 2020 | 1 | 2 | 9 | 9 | 12 | 4 | 3 |  | 40 |
| Total | 10 | 50 | 70 | 57 | 59 | 17 | 13 | 4 | 280 |

**Table B. Number of clusters identified in unadjusted and poverty adjusted analyses.**

The table below shows the number of clusters detected in the unadjusted and poverty-adjusted analyses by disease and year. This information is also shown in Figure 1 (A-B) through a visual format in each map.

| Number of Clusters Identified | | |
| --- | --- | --- |
| Disease | Unadjusted | Poverty-adjusted |
| TB 2019 | 8 | 15 |
| TB 2020 | 5 | 16 |
| HIV 2019 | 7 | 36 |
| HIV 2020 | 10 | 40 |
| Influenza 2020 | 8 | 2 |
| Influenza 2021 | 11 | 2 |
| COVID-19 2021 | 11 | 100 |
| COVID-19 2022 | 12 | 69 |
