# Supplementary material for: Concurrent disease burden from multiple infectious diseases and the influence of social determinants in the contiguous United States
Source: PLoS One. 2024 Sep 4;19(9):e0293431. doi: 10.1371/journal.pone.0293431 (PMC11373817; doi:10.1371/journal.pone.0293431)
Supplement: S3 File — Included in the tables are the county name, state, p-value, expected number of cases, observed number of cases, the relative risk for the disease, and the county population. (DOCX) [file pone.0293431.s003.docx]

**Supporting Information**

**S3 File**

The outputs from the unadjusted scan statistic for each of the studied diseases (COVID-19, HIV, Influenza, and TB) are shown by year in Tables C-AF.

**Table C. COVID-19 2021.**

| *County* | *State* | *P-Value* | *Expected* | *Observed* | *Relative Risk* | *Population* |
| --- | --- | --- | --- | --- | --- | --- |
| Clark | NV | 0.00 | 1169725 | 1532136 | 1.34 | 19344744 |
| Franklin | ID | 0.00 | 222519 | 308940 | 1.39 | 3679983 |
| Gaines | TX | 0.00 | 187413 | 307105 | 1.65 | 3099411 |
| Grady | GA | 0.00 | 122914 | 148635 | 1.21 | 2032726 |
| Kittson | MN | 0.00 | 1807804 | 2419026 | 1.38 | 29897197 |
| Lauderdale | TN | 0.00 | 1590991 | 1978919 | 1.27 | 26311580 |
| McCormick | SC | 0.00 | 250602 | 272737 | 1.09 | 4144428 |
| Miami-Dade | FL | 0.00 | 161011 | 298872 | 1.87 | 2662777 |
| New York | NY | 0.00 | 95349 | 436692 | 4.66 | 1576876 |
| Westmoreland | PA | 0.00 | 408 | 19451 | 47.74 | 6745 |
| Yuma | AZ | 0.00 | 601 | 27812 | 46.33 | 9941 |

**Table D. COVID-19 2022.**

| *County* | *State* | *P-Value* | *Expected* | *Observed* | *Relative Risk* | *Population* |
| --- | --- | --- | --- | --- | --- | --- |
| Fairfield | SC | 0.00 | 1151846 | 1307900 | 1.14 | 7019067 |
| Franklin | FL | 0.00 | 2880031 | 3021002 | 1.05 | 17550209 |
| Garfield | UT | 0.00 | 2618705 | 2926431 | 1.12 | 15957751 |
| Hamilton | TN | 0.00 | 10579 | 71335 | 6.75 | 64468 |
| Hansford | TX | 0.00 | 234476 | 300806 | 1.28 | 1428841 |
| Miami-Dade | FL | 0.00 | 436968 | 847746 | 1.96 | 2662777 |
| Neosho | KS | 0.00 | 55467 | 68498 | 1.24 | 338002 |
| New York | NY | 0.00 | 258769 | 1594682 | 6.32 | 1576876 |
| Scott | IN | 0.00 | 4495485 | 5062029 | 1.14 | 27394393 |
| Traill | ND | 0.00 | 3442998 | 3879004 | 1.14 | 20980789 |
| Westmoreland | PA | 0.00 | 1107 | 59832 | 54.11 | 6745 |
| Yuma | AZ | 0.00 | 1631 | 44644 | 27.39 | 9941 |

**Table E. HIV 2019.**

| *County* | *State* | *P-Value* | *Expected* | *Observed* | *Relative Risk* | *Population* |
| --- | --- | --- | --- | --- | --- | --- |
| Anne Arundel | MD | 0.00 | 8345 | 33653 | 4.14 | 2787800 |
| Bronx | NY | 0.00 | 4245 | 27967 | 6.75 | 1418207 |
| Jackson | LA | 0.00 | 66903 | 96338 | 1.49 | 22350956 |
| Kings | NY | 0.00 | 25114 | 85474 | 3.63 | 8389976 |
| Lake | FL | 0.00 | 95007 | 171462 | 1.97 | 31740017 |
| Los Angeles | CA | 0.00 | 30050 | 49720 | 1.69 | 10039107 |
| San Francisco | CA | 0.00 | 2639 | 12006 | 4.59 | 881549 |

**Table F. HIV 2020.**

| *County* | *State* | *P-Value* | *Expected* | *Observed* | *Relative Risk* | *Population* |
| --- | --- | --- | --- | --- | --- | --- |
| Acadia | LA | 0.00 | 35817 | 54939 | 1.56 | 11406018 |
| Anne Arundel | MD | 0.00 | 8879 | 33149 | 3.82 | 2827595 |
| Bronx | NY | 0.00 | 4605 | 27676 | 6.15 | 1466438 |
| Dallas | TX | 0.00 | 8199 | 18983 | 2.34 | 2610857 |
| Fairfield | SC | 0.00 | 131 | 2795 | 21.39 | 41716 |
| Kings | NY | 0.00 | 20705 | 84765 | 4.37 | 6593451 |
| Lake | FL | 0.00 | 100701 | 174166 | 1.88 | 320068365 |
| Los Angeles | CA | 0.00 | 31368 | 50243 | 1.63 | 9989165 |
| San Francisco | CA | 0.00 | 2732 | 11803 | 4.63 | 870014 |
| Tallahatchie | FL | 0.00 | 5961 | 9029 | 1.52 | 1898165 |

**Table G. INFLUENZA 2020.**

| *County* | *State* | *P-Value* | *Expected* | *Observed* | *Relative Risk* | *Population* |
| --- | --- | --- | --- | --- | --- | --- |
| Boone | MO | 0.00 | 24655 | 342879 | 14.07 | 180463 |
| Cameron | TX | 0.00 | 530782 | 6583267 | 15.88 | 3885002 |
| Coffee | GA | 0.00 | 2793568 | 6903966 | 2.95 | 20447232 |
| Dare | NC | 0.00 | 509598 | 786579 | 1.56 | 3729953 |
| Lee | FL | 0.00 | 131088 | 3058636 | 26.06 | 959487 |
| Mississippi | AR | 0.00 | 183234 | 423419 | 2.33 | 1341164 |
| Sacramento | CA | 0.00 | 296594 | 1128860 | 3.92 | 2170887 |
| San Miguel | NM | 0.00 | 141377 | 481635 | 3.45 | 1034795 |

**Table H. INFLUENZA 2021.**

| *County* | *State* | *P-Value* | *Expected* | *Observed* | *Relative Risk* | *Population* |
| --- | --- | --- | --- | --- | --- | --- |
| Bernalillo | NM | 0.00 | 529925 | 4808177 | 9.32 | 679121 |
| Boone | MO | 0.00 | 140817 | 808474 | 5.77 | 180463 |
| Buncombe | NC | 0.00 | 203810 | 726111 | 3.57 | 261191 |
| Champaign | IL | 0.00 | 163622 | 4474763 | 28.10 | 209689 |
| Clark | NV | 0.00 | 1768740 | 5394781 | 3.12 | 2266715 |
| Fairfield | CT | 0.00 | 9197204 | 14333436 | 1.61 | 11786605 |
| Hidalgo | TX | 0.00 | 4503063 | 22858430 | 5.75 | 5770865 |
| Montgomery | OH | 0.00 | 414881 | 2892376 | 7.08 | 531687 |
| Newberry | SC | 0.00 | 354416 | 728316 | 2.06 | 454199 |
| Virginia Beach | VA | 0.00 | 898311 | 2973012 | 3.35 | 1151224 |
| Volusia | FL | 0.00 | 12289034 | 39032782 | 3.87 | 15748916 |

**Table I. TB 2019.**

| *County* | *State* | *P-Value* | *Expected* | *Observed* | *Relative Risk* | *Population* |
| --- | --- | --- | --- | --- | --- | --- |
| Bronx | NY | 0.00 | 32 | 88 | 2.81 | 1417207 |
| Calhoun | TX | 0.00 | 340 | 736 | 2.29 | 15272882 |
| Dallas | TX | 0.00 | 59 | 135 | 2.33 | 2635516 |
| Falls Church | VA | 0.00 | 126 | 283 | 2.29 | 5679585 |
| King | WA | 0.00 | 50 | 132 | 2.66 | 2252782 |
| Kings | NY | 0.00 | 293 | 751 | 2.73 | 13181126 |
| Monroe | FL | 0.00 | 71 | 134 | 1.91 | 3176070 |
| Ventura | CA | 0.00 | 719 | 1658 | 2.68 | 32296530 |

**Table J. TB 2020.**

| *County* | *State* | *P-Value* | *Expected* | *Observed* | *Relative Risk* | *Population* |
| --- | --- | --- | --- | --- | --- | --- |
| Bronx | NY | 0.00 | 26 | 80 | 3.08 | 1466438 |
| Fairfax | VA | 0.00 | 94 | 201 | 2.17 | 5287715 |
| Hudson | NJ | 0.00 | 245 | 596 | 2.59 | 13708337 |
| Montgomery | TX | 0.00 | 98 | 287 | 2.99 | 5461455 |
| Santa Barbara | CA | 0.00 | 576 | 1440 | 3.04 | 32217293 |
