# Supplementary material for: Concurrent disease burden from multiple infectious diseases and the influence of social determinants in the contiguous United States
Source: PLoS One. 2024 Sep 4;19(9):e0293431. doi: 10.1371/journal.pone.0293431 (PMC11373817; doi:10.1371/journal.pone.0293431)
Supplement: S4 File — Included in the table are the county name, state, p-value, expected number of cases, observed number of cases, the relative risk for the disease, and the county population. (DOCX) [file pone.0293431.s004.docx]

**Supporting Information**

**S4 File**

**Tables K-M.** The following tables compare the counties that had a high relative risk for the same disease in consecutive years in the unadjusted analysis. Included in the table are the county name, state, p-value, expected number of cases, observed number of cases, the relative risk for the disease, and the county population.

**Table K. COVID-19 2021 & 2022.**

| *Disease* | *County* | *State* | *P-Value* | *Expected* | *Observed* | *Relative Risk* | *Population* |
| --- | --- | --- | --- | --- | --- | --- | --- |
| COVID-19 2021 | Miami-Dade | FL | 0.00 | 161011 | 298872 | 1.87 | 2662777 |
| COVID-19 2022 | Miami-Dade | FL | 0.00 | 436968 | 847746 | 1.96 | 2662777 |
| COVID-19 2021 | New York | NY | 0.00 | 95349 | 436692 | 4.66 | 1576876 |
| COVID-19 2022 | New York | NY | 0.00 | 258769 | 1594682 | 6.32 | 1576876 |
| COVID-19 2021 | Westmoreland | PA | 0.00 | 408 | 19451 | 47.74 | 6745 |
| COVID-19 2022 | Westmoreland | PA | 0.00 | 1107 | 59832 | 54.11 | 6745 |
| COVID-19 2021 | Yuma | AZ | 0.00 | 601 | 27812 | 46.33 | 9941 |
| COVID-19 2022 | Yuma | AZ | 0.00 | 1631 | 44644 | 27.39 | 9941 |

**Table L. HIV 2019 & 2020.**

| *Disease* | *County* | *State* | *P-Value* | *Expected* | *Observed* | *Relative Risk* | *Population* |
| --- | --- | --- | --- | --- | --- | --- | --- |
| HIV 2019 | Anne Arundel | MD | 0.00 | 8345 | 33653 | 4.14 | 2787800 |
| HIV 2020 | Anne Arundel | MD | 0.00 | 8879 | 33149 | 3.82 | 2827595 |
| HIV 2019 | Lake | FL | 0.00 | 95006 | 171462 | 1.97 | 31740017 |
| HIV 2020 | Lake | FL | 0.00 | 100700 | 174166 | 1.88 | 32068365 |
| HIV 2019 | Los Angeles | CA | 0.00 | 30050 | 49720 | 1.69 | 10039107 |
| HIV 2020 | Los Angeles | CA | 0.00 | 31368 | 50243 | 1.63 | 9989165 |
| HIV 2019 | San Francisco | CA | 0.00 | 2639 | 12006 | 4.59 | 881549 |
| HIV 2020 | San Francisco | CA | 0.00 | 2732 | 11803 | 4.36 | 870014 |

**Table M. INFLUENZA 2019 & 2020.**

| *Disease* | *County* | *State* | *P-Value* |  | *Expected* | *Observed* | *Relative Risk* | *Population* |
| --- | --- | --- | --- | --- | --- | --- | --- | --- |
| INFLUENZA 2020 | Boone | MO | 0.00 |  | 24655 | 342879 | 14.07 | 180463 |
| INFLUENZA 2021 | Boone | MO | 0.00 |  | 140817 | 808474 | 5.77 | 180463 |
