# Supplementary material for: Concurrent disease burden from multiple infectious diseases and the influence of social determinants in the contiguous United States
Source: PLoS One. 2024 Sep 4;19(9):e0293431. doi: 10.1371/journal.pone.0293431 (PMC11373817; doi:10.1371/journal.pone.0293431)
Supplement: S5 File — Included in the table are the county name, state, p-value, expected number of cases, observed number of cases, the relative risk for the disease, and the county population. (DOCX) [file pone.0293431.s005.docx]

**Supporting Information**

**S5 File**

**Tables N-P.** The following tables list the counties with a high relative risk for two different diseases in the unadjusted analysis. Included in the table are the county name, state, p-value, expected number of cases, observed number of cases, the relative risk for the disease, and the county population.

**Table N. COVID-19 & HIV.**

| *Disease* | *County* | *State* | *P-Value* | *Observed* | *Expected* | *Relative Risk* | *Population* |
| --- | --- | --- | --- | --- | --- | --- | --- |
| COVID-19 2022 | Fairfield | SC | 0.00 | 1307900 | 1151846 | 1.14 | 7019067 |
| HIV 2020 | Fairfield | SC | 0.00 | 2795 | 131 | 21.39 | 41716 |

**Table O. COVID-19 & INFLUENZA.**

| *Disease* | *County* | *State* | *P-Value* | *Observed* | *Expected* | *Relative Risk* | *Population* |
| --- | --- | --- | --- | --- | --- | --- | --- |
| COVID-19 2021 | Clark | NV | 0.00 | 1532136 | 1169725 | 1.34 | 19344744 |
| INFLUENZA 2021 | Clark | NV | 0.00 | 5394781 | 1768740 | 3.12 | 2266715 |

**Table P. HIV & TB.**

| *Disease* | *County* | *State* | *P-Value* | *Observed* | *Expected* | *Relative Risk* | *Population* |
| --- | --- | --- | --- | --- | --- | --- | --- |
| HIV 2019 | Bronx | NY | 0.00 | 27967 | 4245 | 6.75 | 1418207 |
| HIV 2020 | Bronx | NY | 0.00 | 27676 | 4605 | 6.15 | 1466438 |
| TB 2019 | Bronx | NY | 0.00 | 88 | 32 | 2.81 | 1418207 |
| TB 2020 | Bronx | NY | 0.00 | 80 | 26 | 3.08 | 1466438 |
| HIV 2020 | Dallas | TX | 0.00 | 18983 | 8199 | 2.34 | 2610957 |
| TB 2019 | Dallas | TX | 0.00 | 135 | 59 | 2.33 | 2635516 |
| HIV 2019 | Kings | NY | 0.00 | 85474 | 25114 | 3.63 | 8389976 |
| HIV 2020 | Kings | NY | 0.00 | 84765 | 20705 | 4.37 | 6593451 |
| TB 2019 | Kings | NY | 0.00 | 751 | 293 | 2.73 | 13181126 |
