# Supplementary material for: Concurrent disease burden from multiple infectious diseases and the influence of social determinants in the contiguous United States
Source: PLoS One. 2024 Sep 4;19(9):e0293431. doi: 10.1371/journal.pone.0293431 (PMC11373817; doi:10.1371/journal.pone.0293431)
Supplement: S6 File — Included in the table are the county name, state, p-value, expected number of cases, observed number of cases, the relative risk for the disease, the county population, the number of individuals below the poverty line in the county, and the percent of the county population that is 125% below the US poverty line. (DOCX) [file pone.0293431.s006.docx]

**Supporting Information**

**S6 File**

The following tables list the counties that were at a high relative risk for each of the diseases, COVID-19, HIV, Influenza, and TB in the years 2019-2022, adjusted for the percent of the county 125% below the poverty line. Included in the table are the county name, state, p-value, expected number of cases, observed number of cases, the relative risk for the disease, the county population, the number of individuals below the poverty line in the county, and the percent of the county population that is 125% below the US poverty line.

**Table Q. COVID-19 2021.**

| *County* | *State* | *P-Value* | *Expected* | *Observed* | *Relative Risk* | *Population* | *POV* | *125percbelow* |
| --- | --- | --- | --- | --- | --- | --- | --- | --- |
| Adair | MO | 0.00 | 660 | 1764 | 2.67 | 25246 | 6832 | 29.87 |
| Adair | IA | 0.00 | 1221 | 1764 | 1.44 | 7477 | 997 | 14.48 |
| Auglaize | OH | 0.00 | 2135 | 3642 | 1.71 | 46411 | 4898 | 10.88 |
| Austin | TX | 0.00 | 759 | 1037 | 1.37 | 30109 | 5403 | 18.26 |
| Baldwin | GA | 0.00 | 2010 | 3245 | 1.61 | 43773 | 11540 | 28.28 |
| Benton | TN | 0.00 | 174 | 753 | 4.32 | 15812 | 3973 | 24.86 |
| Bond | IL | 0.46 | 1400 | 1554 | 1.11 | 16663 | 2743 | 18.62 |
| Boundary | ID | 0.00 | 333 | 569 | 1.71 | 12113 | 2797 | 23.23 |
| Boyle | KY | 0.00 | 1203 | 2162 | 1.80 | 30701 | 5345 | 19.82 |
| Buena Vista | IA | 0.00 | 2486 | 3906 | 1.57 | 37109 | 3023 | 15.96 |
| Carlton | MN | 0.89 | 2414 | 2595 | 1.08 | 36156 | 4967 | 14.64 |
| Chambers | TX | 0.00 | 2003 | 2627 | 1.31 | 47051 | 6957 | 16.47 |
| Chattooga | GA | 0.00 | 1134 | 1842 | 1.62 | 24921 | 6543 | 28.73 |
| Claiborne | MS | 0.00 | 976 | 2237 | 2.29 | 16347 | 3686 | 44.81 |
| Clearwater | MN | 0.00 | 567 | 1224 | 2.16 | 13954 | 1736 | 19.94 |
| Columbia | AR | 0.00 | 1999 | 4102 | 2.05 | 74020 | 6505 | 29.85 |
| Concho | TX | 0.00 | 122 | 210 | 1.72 | 3319 | 438 | 21.32 |
| Cooper | MO | 0.00 | 15430 | 18171 | 1.18 | 391056 | 2748 | 17.23 |
| Crowley | CO | 0.00 | 860 | 1660 | 1.93 | 5893 | 1375 | 30.21 |
| Dade | MO | 0.00 | 521 | 828 | 1.59 | 7586 | 1863 | 25.09 |
| Davison | SD | 0.00 | 1357 | 2718 | 2.00 | 19936 | 3105 | 16.33 |
| Dearborn | IN | 0.00 | 2407 | 3885 | 1.61 | 50726 | 5735 | 11.73 |
| Decatur | TN | 0.00 | 114 | 231 | 2.03 | 11402 | 2673 | 23.38 |
| Deer Lodge | MT | 0.00 | 626 | 847 | 1.35 | 9413 | 2676 | 31.19 |
| Dickens | TX | 0.00 | 68 | 130 | 1.93 | 1760 | 462 | 22.27 |
| Doddridge | WV | 0.00 | 142 | 257 | 1.81 | 7786 | 1611 | 20.97 |
| Edgar | IL | 0.00 | 1159 | 1344 | 1.16 | 16755 | 2600 | 15.33 |
| Effingham | GA | 0.00 | 1620 | 2722 | 1.68 | 65150 | 6975 | 11.32 |
| Elliott | KY | 0.00 | 449 | 784 | 1.75 | 7334 | 2023 | 34.21 |
| Erie | OH | 0.00 | 2735 | 4912 | 1.80 | 75429 | 11853 | 16.18 |
| Falls | TX | 0.00 | 706 | 1199 | 1.70 | 16961 | 4790 | 30.84 |
| Faribault | MN | 0.00 | 740 | 911 | 1.23 | 13888 | 1932 | 14.48 |
| Gila | AR | 0.00 | 2663 | 4552 | 1.71 | 53306 | 13883 | 26.28 |
| Gordon | GA | 0.00 | 4265 | 4962 | 1.16 | 57696 | 11962 | 20.99 |
| Grady | GA | 0.00 | 726 | 1341 | 1.85 | 26221 | 5712 | 23.43 |
| Grayson | KY | 0.00 | 616 | 793 | 1.29 | 26410 | 7771 | 29.89 |
| Greensville | VA | 0.00 | 516 | 1019 | 1.98 | 11393 | 1491 | 18.45 |
| Greer | OK | 0.00 | 326 | 580 | 1.78 | 7953 | 1235 | 25.93 |
| Hansford | TX | 0.00 | 274 | 617 | 2.26 | 5255 | 1409 | 26.28 |
| Hardy | WV | 0.00 | 2769 | 3058 | 1.10 | 58495 | 2576 | 18.88 |
| Hockley | TX | 0.00 | 1370 | 1906 | 1.39 | 21490 | 4565 | 20.60 |
| Hood River | OR | 0.00 | 1142 | 1744 | 1.53 | 34102 | 2062 | 8.93 |
| Iosco | MI | 0.00 | 512 | 1104 | 2.16 | 25191 | 5127 | 20.64 |
| Iowa | IA | 0.00 | 1396 | 1746 | 1.25 | 16679 | 1821 | 11.46 |
| Irwin | GA | 0.00 | 321 | 674 | 2.10 | 9643 | 2712 | 29.37 |
| Jeff Davis | TX | 0.00 | 48 | 108 | 2.25 | 1978 | 7563 | 24.48 |
| Kerr | TX | 0.00 | 1721 | 2380 | 1.38 | 52676 | 8864 | 17.50 |
| Knott | KY | 0.00 | 284 | 738 | 2.60 | 14160 | 5821 | 40.70 |
| La Salle | TX | 0.00 | 419 | 667 | 1.59 | 6642 | 1561 | 23.77 |
| Lee | KY | 0.00 | 487 | 1043 | 2.14 | 7360 | 2807 | 42.39 |
| Louisa | IA | 0.95 | 827 | 931 | 1.13 | 10806 | 1406 | 12.82 |
| Macon | AL | 0.00 | 804 | 1400 | 1.74 | 19476 | 4822 | 29.84 |
| Manistee | MI | 0.00 | 419 | 609 | 1.45 | 25063 | 3494 | 15.04 |
| Marengo | AL | 0.00 | 1977 | 2888 | 1.46 | 31883 | 5194 | 27.51 |
| Martin | TX | 0.00 | 468 | 657 | 1.40 | 5250 | 792 | 14.09 |
| Mississippi | MO | 0.00 | 263 | 1139 | 4.32 | 12537 | 4083 | 35.18 |
| Mono | CA | 0.00 | 450 | 868 | 1.93 | 13206 | 1943 | 13.64 |
| Montgomery | AR | 0.00 | 1322 | 2316 | 1.75 | 8459 | 2138 | 24.26 |
| Montrose | CO | 0.00 | 1300 | 2134 | 1.64 | 42798 | 6851 | 16.37 |
| Morrow | OH | 0.00 | 967 | 1834 | 1.90 | 34964 | 4390 | 12.60 |
| Murray | MN | 0.00 | 4104 | 5160 | 1.26 | 39756 | 925 | 11.45 |
| Nacogdoches | TX | 0.00 | 1267 | 3029 | 2.39 | 64624 | 17878 | 30.04 |
| Nicollet | MN | 0.00 | 2309 | 3094 | 1.34 | 49292 | 3907 | 12.28 |
| Nodaway | MO | 0.00 | 1496 | 2348 | 1.57 | 21218 | 4709 | 24.59 |
| Nolan | TX | 0.00 | 999 | 1248 | 1.25 | 14721 | 3563 | 24.33 |
| Northampton | VA | 0.09 | 912 | 1052 | 1.15 | 12232 | 2494 | 21.64 |
| Nottoway | VA | 0.00 | 873 | 1176 | 1.35 | 15614 | 3114 | 22.96 |
| Ozaukee | WI | 0.00 | 5962 | 7115 | 1.19 | 91569 | 6664 | 7.65 |
| Pawnee | KS | 0.00 | 13868 | 20491 | 1.48 | 201367 | 687 | 12.53 |
| Payette | ID | 0.00 | 1500 | 2064 | 1.38 | 25589 | 3715 | 15.89 |
| Pembina | ND | 0.00 | 419 | 699 | 1.67 | 6827 | 833 | 12.58 |
| Pemiscot | MO | 0.00 | 487 | 1285 | 2.64 | 15585 | 6351 | 39.51 |
| Pontotoc | MS | 0.00 | 1520 | 3135 | 2.06 | 31206 | 7038 | 22.23 |
| Pope | IL | 0.00 | 770 | 1089 | 1.41 | 17869 | 1065 | 26.50 |
| Richland | IL | 0.00 | 602 | 875 | 1.45 | 15803 | 2397 | 15.56 |
| Richmond | VA | 0.00 | 651 | 819 | 1.26 | 8920 | 1976 | 27.20 |
| Ringgold | IA | 0.00 | 126 | 367 | 2.92 | 4639 | 941 | 19.98 |
| Roosevelt | MT | 0.00 | 424 | 1527 | 3.60 | 10780 | 4139 | 38.87 |
| Schoharie | NY | 0.65 | 484 | 572 | 1.18 | 29720 | 4685 | 15.70 |
| Scott | IN | 0.00 | 240 | 327 | 1.36 | 24336 | 5539 | 24.19 |
| Scotts Bluff | NE | 0.00 | 2077 | 4129 | 1.99 | 36002 | 6240 | 17.89 |
| Seminole | OK | 0.00 | 322 | 688 | 2.14 | 23508 | 7058 | 29.52 |
| Sevier | AR | 0.00 | 379 | 1356 | 3.58 | 15778 | 4457 | 26.57 |
| Simpson | MS | 0.00 | 10424 | 14827 | 1.42 | 238704 | 6898 | 26.29 |
| Smyth | VA | 0.00 | 1500 | 1857 | 1.24 | 29730 | 7251 | 24.36 |
| Stanly | NC | 0.00 | 1774 | 4331 | 2.44 | 62609 | 10197 | 16.95 |
| Starke | IN | 0.00 | 990 | 1564 | 1.58 | 23356 | 4953 | 21.68 |
| Ste. Genevieve | MO | 0.00 | 1153 | 1430 | 1.24 | 18476 | 2336 | 13.30 |
| Talbot | GA | 0.00 | 388 | 1185 | 3.05 | 5731 | 1829 | 29.46 |
| Taylor | WI | 0.00 | 405 | 547 | 1.35 | 19897 | 2918 | 14.60 |
| Tipton | IN | 0.00 | 819 | 1105 | 1.35 | 15365 | 1932 | 12.99 |
| Toole | MT | 0.00 | 471 | 673 | 1.43 | 4964 | 600 | 14.19 |
| Towns | GA | 0.00 | 632 | 819 | 1.30 | 12539 | 1874 | 17.12 |
| Trempealeau | WI | 0.00 | 2521 | 3064 | 1.22 | 30776 | 3648 | 12.57 |
| Treutlen | GA | 0.00 | 2466 | 4707 | 1.91 | 37697 | 2330 | 36.41 |
| Union | KY | 0.00 | 395 | 537 | 1.36 | 13646 | 2974 | 20.90 |
| Warren | VA | 0.00 | 259 | 547 | 2.11 | 40782 | 5216 | 13.35 |
| Wasatch | UT | 0.00 | 2237 | 3196 | 1.43 | 35032 | 2720 | 8.26 |
| Washakie | WY | 0.00 | 279 | 780 | 2.79 | 7658 | 1080 | 13.95 |
| Woodford | KY | 0.00 | 690 | 1253 | 1.82 | 26892 | 3336 | 12.81 |

**Table R. COVID-19 2022.**

| *County* | *State* | *P-Value* | *Expected* | *Observed* | *Relative Risk* | *Population* | *POV* | *125percbelow* |
| --- | --- | --- | --- | --- | --- | --- | --- | --- |
| Abbeville | SC | 0.00 | 12196 | 14139 | 1.16 | 324955 | 5761 | 24.13 |
| Adams | ID | 0.00 | 41161 | 95323 | 2.32 | 4424 | 792 | 19.01 |
| Addison | VT | 0.00 | 2068 | 2548 | 1.23 | 37292 | 3507 | 10.36 |
| Allen | KS | 0.00 | 33613 | 75061 | 2.23 | 12537 | 2600 | 21.60 |
| Allen | LA | 0.00 | 45464 | 75061 | 1.65 | 22750 | 4210 | 20.16 |
| Armstrong | TX | 0.00 | 8382 | 11982 | 1.43 | 1847 | 154 | 8.23 |
| Benton | TN | 0.00 | 9890 | 46855 | 4.74 | 15812 | 3973 | 24.86 |
| Calhoun | FL | 0.00 | 11447 | 23411 | 2.05 | 13615 | 2918 | 23.74 |
| Calhoun | AR | 0.00 | 8551 | 23411 | 2.74 | 4725 | 945 | 18.79 |
| Clay | IA | 0.00 | 1329 | 2658 | 2.00 | 16333 | 2239 | 14.16 |
| Clearwater | MN | 0.00 | 1880 | 2920 | 1.55 | 13954 | 1736 | 19.94 |
| Columbia | WI | 0.00 | 2617 | 3627 | 1.39 | 58510 | 5216 | 9.35 |
| Concho | TX | 0.00 | 512 | 793 | 1.55 | 3319 | 438 | 21.32 |
| Crowley | CO | 0.00 | 1758 | 2499 | 1.42 | 5893 | 1375 | 30.21 |
| Dickey | ND | 0.00 | 942 | 1191 | 1.26 | 4959 | 430 | 9.27 |
| Douglas | SD | 0.00 | 27989 | 52417 | 1.87 | 2824 | 355 | 12.64 |
| Douglas | MO | 0.00 | 25588 | 52417 | 2.05 | 11592 | 2867 | 21.80 |
| Edmunds | SD | 0.00 | 16921 | 21757 | 1.29 | 53449 | 491 | 13.05 |
| Effingham | GA | 0.00 | 5891 | 9250 | 1.57 | 65150 | 6975 | 11.32 |
| Erie | OH | 0.00 | 72786 | 151262 | 2.08 | 75429 | 11853 | 16.18 |
| Frederick | VA | 0.00 | 26018 | 30646 | 1.18 | 91766 | 7455 | 8.64 |
| Gila | AZ | 0.00 | 8044 | 12274 | 1.53 | 53306 | 13883 | 26.28 |
| Hancock | KY | 0.00 | 34519 | 36223 | 1.05 | 315868 | 1694 | 19.57 |
| Hancock | GA | 0.00 | 30507 | 55255 | 1.81 | 81397 | 1806 | 29.62 |
| Hockley | TX | 0.00 | 3407 | 4668 | 1.37 | 21490 | 4565 | 20.60 |
| Jackson | MN | 0.00 | 5873 | 10637 | 1.81 | 9969 | 1080 | 11.14 |
| Jeff Davis | TX | 0.00 | 1030 | 3028 | 2.94 | 1978 | 676 | 30.95 |
| Jefferson | IN | 0.00 | 68318 | 126583 | 1.85 | 33075 | 5403 | 18.18 |
| Jefferson | MS | 0.00 | 53589 | 126583 | 2.37 | 7254 | 2309 | 34.99 |
| Jefferson | NE | 0.00 | 87528 | 147152 | 1.68 | 21509 | 1036 | 14.89 |
| Johnson | WY | 0.00 | 3027 | 4902 | 1.62 | 8459 | 1406 | 16.73 |
| Juniata | PA | 0.94 | 3610 | 3826 | 1.06 | 23451 | 3273 | 13.44 |
| Kerr | TX | 0.00 | 5346 | 7358 | 1.38 | 52676 | 8864 | 17.50 |
| Knox | TX | 0.00 | 4651 | 8838 | 1.90 | 3361 | 653 | 18.38 |
| Lake | CO | 0.00 | 4070 | 7239 | 1.78 | 7409 | 1309 | 16.92 |
| Lee | KY | 0.00 | 9657 | 26975 | 2.79 | 7360 | 2807 | 42.39 |
| Lee | TX | 0.00 | 13220 | 26975 | 2.04 | 17499 | 2891 | 17.27 |
| Madison | TX | 0.00 | 36453 | 57750 | 1.58 | 13445 | 1972 | 17.22 |
| Marshall | OK | 0.00 | 38647 | 56464 | 1.46 | 25564 | 3685 | 22.36 |
| Marshall | IA | 0.00 | 14114 | 19507 | 1.38 | 40059 | 6240 | 16.23 |
| Mississippi | MO | 0.00 | 1911 | 9527 | 4.99 | 12537 | 4083 | 35.18 |
| Mitchell | IA | 0.00 | 2541 | 3634 | 1.43 | 10582 | 1230 | 11.91 |
| Montgomery | GA | 0.00 | 15446 | 37879 | 2.45 | 8577 | 1901 | 23.02 |
| Montrose | CO | 0.00 | 4119 | 7030 | 1.71 | 42798 | 6851 | 16.37 |
| Moore | TX | 0.00 | 10421 | 15215 | 1.46 | 21273 | 3111 | 14.83 |
| Morgan | TN | 0.00 | 5745 | 23831 | 4.15 | 21020 | 5408 | 29.06 |
| Murray | MN | 0.01 | 13028 | 13620 | 1.05 | 30351 | 925 | 11.45 |
| Nicollet | MN | 0.00 | 7232 | 8525 | 1.18 | 49292 | 3907 | 12.28 |
| Orleans | NY | 0.00 | 38306 | 55985 | 1.46 | 40236 | 6878 | 18.18 |
| Ozaukee | WI | 0.00 | 14764 | 15937 | 1.08 | 91569 | 6664 | 7.65 |
| Perkins | SD | 0.00 | 77004 | 135334 | 1.76 | 64332 | 440 | 14.94 |
| Pickaway | OH | 0.00 | 174523 | 234581 | 1.35 | 1691745 | 8871 | 16.59 |
| Pike | IN | 0.00 | 4567 | 5433 | 1.19 | 12233 | 1686 | 13.96 |
| Pope | IL | 0.00 | 28389 | 49153 | 1.73 | 67289 | 1065 | 26.50 |
| Pope | MN | 0.00 | 9302 | 12976 | 1.40 | 11309 | 1235 | 11.32 |
| Richland | IL | 0.00 | 31467 | 34848 | 1.11 | 105832 | 2397 | 15.56 |
| Richmond | VA | 0.00 | 9032 | 38075 | 4.22 | 8920 | 1976 | 27.20 |
| Roberts | SD | 0.62 | 1610 | 1769 | 1.10 | 10257 | 2797 | 27.66 |
| Saline | MO | 0.00 | 9261 | 20569 | 2.22 | 23319 | 4591 | 21.30 |
| Sawyer | WI | 0.99 | 3317 | 3513 | 1.06 | 18107 | 3350 | 20.78 |
| Schoharie | NY | 0.00 | 2986 | 3470 | 1.16 | 29720 | 4685 | 15.70 |
| Seminole | OK | 0.00 | 14007 | 71009 | 5.08 | 23508 | 7058 | 29.52 |
| Shelby | IA | 0.00 | 24371 | 41228 | 1.69 | 11699 | 1457 | 12.86 |
| Southampton | VA | 0.00 | 50989 | 70523 | 1.38 | 95997 | 1984 | 12.20 |
| Stanly | NC | 0.00 | 5069 | 13128 | 2.59 | 62609 | 10197 | 16.95 |
| Trempealeau | WI | 0.00 | 4929 | 6192 | 1.26 | 30776 | 3648 | 12.57 |
| Union | KY | 0.00 | 4549 | 6594 | 1.45 | 13646 | 2974 | 20.90 |
| Wexford | MI | 0.00 | 4416 | 5056 | 1.15 | 33676 | 6488 | 19.65 |
| Windsor | VT | 0.00 | 4418 | 5021 | 1.14 | 57700 | 6832 | 12.56 |

**Table S. HIV 2019.**

| *County* | *State* | *P-Value* | *Expected* | *Observed* | *Relative Risk* | *Population* | *POV* | *125percbelow* |
| --- | --- | --- | --- | --- | --- | --- | --- | --- |
| Allen | LA | 0.01 | 119 | 176 | 1.48 | 22750 | 4210 | 20.16 |
| Allendale | SC | 0.01 | 16 | 40 | 2.50 | 7990 | 2807 | 36.10 |
| Austin | TX | 0.01 | 28 | 59 | 2.10 | 30109 | 5403 | 18.26 |
| Baldwin | GA | 0.00 | 66 | 129 | 1.95 | 43773 | 11540 | 28.28 |
| Benton | TN | 0.66 | 1 | 7 | 6.20 | 15812 | 3973 | 24.86 |
| Columbia | AR | 0.00 | 51 | 138 | 2.68 | 74020 | 6505 | 29.85 |
| Dillon | SC | 0.00 | 39 | 146 | 3.75 | 28284 | 10596 | 35.28 |
| Doddridge | WV | 0.60 | 1 | 8 | 5.48 | 7786 | 1611 | 20.97 |
| Dooly | GA | 0.01 | 30 | 61 | 2.02 | 11142 | 3133 | 25.88 |
| Dorchester | MD | 0.00 | 78 | 148 | 1.91 | 32508 | 6543 | 20.75 |
| Falls | TX | 0.00 | 18 | 45 | 2.46 | 16961 | 4790 | 30.84 |
| Gila | AZ | 0.54 | 25 | 47 | 1.85 | 53306 | 13883 | 26.28 |
| Grady | GA | 0.25 | 13 | 30 | 2.37 | 26221 | 5712 | 23.43 |
| Greensville | VA | 0.00 | 39 | 79 | 2.01 | 11393 | 1491 | 18.45 |
| Greer | OK | 0.02 | 8 | 26 | 3.06 | 5488 | 1235 | 25.93 |
| Haywood | TN | 0.00 | 31 | 77 | 2.46 | 17795 | 4609 | 26.89 |
| Hood River | OR | 1.00 | 27 | 46 | 1.68 | 34102 | 2062 | 8.93 |
| Huerfano | CO | 0.65 | 3 | 12 | 3.69 | 6810 | 1230 | 18.70 |
| Irwin | GA | 0.00 | 33 | 72 | 2.20 | 9643 | 2712 | 29.37 |
| Karnes | TX | 0.74 | 56 | 86 | 1.52 | 14721 | 2748 | 22.56 |
| Kerr | TX | 0.04 | 35 | 66 | 1.87 | 52676 | 8864 | 17.50 |
| Little River | AR | 0.08 | 8 | 23 | 3.03 | 27749 | 2296 | 19.00 |
| Manistee | MI | 0.12 | 24 | 49 | 2.00 | 117876 | 3494 | 15.04 |
| Mississippi | MO | 0.00 | 15 | 63 | 4.25 | 79271 | 4083 | 35.18 |
| Nacogdoches | TX | 0.00 | 60 | 108 | 1.80 | 64624 | 17878 | 30.04 |
| Nottoway | VA | 0.03 | 70 | 113 | 1.61 | 15614 | 3114 | 22.96 |
| Pacific | WA | 0.79 | 18 | 35 | 1.97 | 23470 | 4083 | 18.80 |
| Pontotoc | MS | 0.89 | 12 | 26 | 2.16 | 31206 | 7038 | 22.23 |
| Richmond | VA | 0.00 | 6 | 28 | 4.86 | 8920 | 1976 | 27.20 |
| San Juan | UT | 0.16 | 36 | 64 | 1.80 | 110842 | 4131 | 27.72 |
| Simpson | MS | 0.00 | 314 | 486 | 1.55 | 238704 | 6898 | 26.29 |
| Stanly | NC | 0.00 | 35 | 97 | 2.75 | 62609 | 10197 | 16.95 |
| Talbot | GA | 0.05 | 7 | 23 | 3.10 | 5731 | 1829 | 29.46 |
| Treutlen | GA | 0.00 | 37 | 96 | 2.60 | 37697 | 2330 | 36.41 |
| Wilkinson | MS | 0.00 | 186 | 315 | 1.70 | 72637 | 2601 | 34.65 |
| Woodford | KY | 0.97 | 9 | 21 | 2.28 | 26892 | 3336 | 12.81 |

**Table T. HIV 2020.**

| *County* | *State* | *P-Value* | *Expected* | *Observed* | *Relative Risk* | *Population* | *POV* | *125percbelow* |
| --- | --- | --- | --- | --- | --- | --- | --- | --- |
| Allen | LA | 0.00 | 126 | 194 | 1.54 | 22750 | 4210 | 20.16 |
| Allendale | SC | 0.01 | 16 | 39 | 2.49 | 7990 | 2807 | 36.10 |
| Austin | TX | 0.02 | 26 | 54 | 2.10 | 30109 | 5403 | 18.26 |
| Benton | TN | 0.66 | 1 | 7 | 6.20 | 15812 | 3973 | 24.86 |
| Columbia | AR | 0.00 | 51 | 141 | 2.78 | 74020 | 6505 | 29.85 |
| Crowley | CO | 0.98 | 11 | 23 | 2.17 | 5893 | 1375 | 30.21 |
| Dillon | SC | 0.00 | 40 | 146 | 3.69 | 28284 | 10596 | 35.28 |
| Doddridge | WV | 0.59 | 1 | 8 | 5.48 | 7786 | 1611 | 20.97 |
| Dooly | GA | 0.00 | 25 | 55 | 2.18 | 11142 | 3133 | 25.88 |
| Dorchester | MD | 0.00 | 78 | 147 | 1.88 | 32508 | 6543 | 20.75 |
| Erie | OH | 0.54 | 59 | 91 | 1.53 | 75429 | 11853 | 16.18 |
| Falls | TX | 0.00 | 19 | 46 | 2.46 | 16961 | 4790 | 30.84 |
| Gila | AZ | 0.23 | 29 | 54 | 1.85 | 53306 | 13883 | 26.28 |
| Grady | GA | 0.33 | 12 | 28 | 2.37 | 26221 | 5712 | 23.43 |
| Greensville | VA | 0.03 | 37 | 69 | 1.88 | 11393 | 1491 | 18.45 |
| Greer | OK | 0.17 | 7 | 20 | 3.06 | 5488 | 1235 | 25.93 |
| Hancock | GA | 0.00 | 76 | 142 | 1.88 | 59257 | 1806 | 29.62 |
| Haywood | TN | 0.00 | 33 | 79 | 2.42 | 17795 | 4609 | 26.89 |
| Hood River | OR | 0.97 | 27 | 46 | 1.70 | 34102 | 2062 | 8.93 |
| Huerfano | CO | 0.20 | 4 | 15 | 3.69 | 6810 | 1230 | 18.70 |
| Irwin | GA | 0.00 | 34 | 76 | 2.20 | 9643 | 2712 | 29.37 |
| Karnes | TX | 0.95 | 56 | 83 | 1.48 | 14721 | 2748 | 22.56 |
| Kerr | TX | 0.03 | 38 | 71 | 1.87 | 52676 | 8864 | 17.50 |
| Little River | AR | 0.05 | 8 | 24 | 3.01 | 27749 | 2296 | 19.00 |
| Manistee | MI | 0.48 | 26 | 48 | 1.85 | 117876 | 3494 | 15.04 |
| Nacogdoches | TX | 0.00 | 61 | 112 | 1.83 | 64624 | 17878 | 30.04 |
| Nottoway | VA | 0.50 | 54 | 85 | 1.56 | 15614 | 3114 | 22.96 |
| Pacific | WA | 0.41 | 17 | 36 | 2.08 | 23470 | 4083 | 18.80 |
| Pleasants | WV | 1.00 | 5 | 13 | 2.61 | 7649 | 978 | 14.08 |
| Pontotoc | MS | 0.89 | 12 | 26 | 2.16 | 31206 | 7038 | 22.23 |
| Richmond | VA | 0.00 | 6 | 28 | 4.86 | 8920 | 1976 | 27.20 |
| Sabine | LA | 0.07 | 9 | 25 | 2.88 | 22118 | 6317 | 26.81 |
| San Juan | UT | 0.08 | 40 | 71 | 1.78 | 110842 | 4131 | 27.72 |
| Scott | MO | 0.00 | 13 | 53 | 3.99 | 55714 | 9317 | 24.67 |
| Simpson | MS | 0.00 | 313 | 480 | 1.53 | 238704 | 6898 | 26.29 |
| Stanly | NC | 0.00 | 39 | 106 | 2.75 | 62609 | 10197 | 16.95 |
| Talbot | GA | 0.08 | 7 | 22 | 3.06 | 5731 | 1829 | 29.46 |
| Treutlen | GA | 0.00 | 33 | 86 | 2.61 | 37697 | 2330 | 36.41 |
| Wilkinson | MS | 0.00 | 98 | 169 | 1.73 | 72637 | 2601 | 34.65 |
| Woodford | KY | 0.97 | 9 | 21 | 2.28 | 26892 | 3336 | 12.81 |

**Table U. INFLUENZA 2020.**

| *County* | *State* | *P-Value* | *Expected* | *Observed* | *Relative Risk* | *Population* | *POV* | *125percbelow* |
| --- | --- | --- | --- | --- | --- | --- | --- | --- |
| Gordon | GA | 0.00 | 7329 | 17388 | 2.37 | 57696 | 11962 | 20.99 |
| Walla Walla | WA | 0.00 | 544 | 1215 | 2.23 | 62574 | 8738 | 15.66 |

**Table V. INFLUENZA 2021.**

| *County* | *State* | *P-Value* | *Expected* | *Observed* | *Relative Risk* | *Population* | *POV* | *125percbelow* |
| --- | --- | --- | --- | --- | --- | --- | --- | --- |
| Boyle | KY | 0.00 | 3677 | 6012 | 1.64 | 30701 | 5345 | 19.82 |
| Gordon | GA | 0.00 | 22470 | 39414 | 1.75 | 57696 | 11962 | 20.99 |

**Table W. TB 2019.**

| *County* | *State* | *P-Value* | *Expected* | *Observed* | *Relative Risk* | *Population* | *POV* | *125percbelow* |
| --- | --- | --- | --- | --- | --- | --- | --- | --- |
| Fulton | IN | 0.00 | 16 | 44 | 2.69 | 20434 | 3907 | 19.84 |
| Fulton | IL | 0.06 | 21 | 44 | 2.11 | 33440 | 5972 | 18.61 |
| Gates | NC | 1.00 | 16 | 29 | 1.80 | 212955 | 2399 | 20.96 |
| Jefferson | MS | 0.94 | 8 | 18 | 2.37 | 7254 | 2309 | 34.99 |
| Jefferson | IN | 0.97 | 13 | 25 | 2.00 | 93436 | 5403 | 18.18 |
| Mineral | WV | 1.00 | 50 | 71 | 1.43 | 814572 | 4551 | 17.44 |
| Monroe | AR | 0.02 | 3 | 15 | 4.45 | 6765 | 1978 | 29.14 |
| Morgan | KY | 0.22 | 6 | 18 | 3.08 | 182375 | 3394 | 29.26 |
| Richmond | VA | 0.00 | 5 | 22 | 4.86 | 8920 | 1976 | 27.20 |
| Stark | ND | 1.00 | 10 | 20 | 2.04 | 52532 | 4036 | 13.23 |
| Toombs | GA | 0.32 | 22 | 43 | 1.93 | 496342 | 6950 | 26.26 |
| Wabash | IL | 0.48 | 9 | 23 | 2.44 | 172913 | 2165 | 19.23 |
| Warren | IN | 0.01 | 1 | 9 | 8.61 | 8413 | 1056 | 13.04 |
| Warren | GA | 1.00 | 3 | 9 | 3.26 | 5205 | 1418 | 27.34 |
| Weakley | TN | 0.00 | 21 | 54 | 2.63 | 274978 | 9071 | 29.32 |

**Table X. TB 2020.**

| *County* | *State* | *P-Value* | *Expected* | *Observed* | *Relative Risk* | *Population* | *POV* | *125percbelow* |
| --- | --- | --- | --- | --- | --- | --- | --- | --- |
| Christian | MO | 0.85 | 10 | 22 | 2.22 | 657012 | 12555 | 14.54 |
| Crittenden | KY | 1.00 | 8 | 17 | 2.23 | 47742 | 1714 | 19.84 |
| Fulton | KY | 0.01 | 10 | 28 | 2.82 | 6501 | 1842 | 32.75 |
| Fulton | IN | 0.04 | 10 | 28 | 2.69 | 20434 | 3907 | 19.84 |
| Fulton | IL | 0.64 | 13 | 28 | 2.11 | 33440 | 5972 | 18.61 |
| Magoffin | KY | 0.27 | 6 | 17 | 3.07 | 194797 | 4458 | 36.56 |
| Marion | TN | 0.82 | 4 | 13 | 2.99 | 28834 | 6543 | 23.17 |
| Monroe | AR | 0.78 | 2 | 8 | 4.45 | 6765 | 1978 | 29.14 |
| Montgomery | AR | 1.00 | 6 | 14 | 2.44 | 147779 | 2138 | 24.26 |
| Richmond | VA | 0.00 | 3 | 16 | 4.86 | 8920 | 1976 | 27.20 |
| Seminole | OK | 0.31 | 1 | 7 | 6.63 | 23508 | 7058 | 29.52 |
| Shelby | IA | 1.00 | 21 | 36 | 1.71 | 11699 | 1457 | 12.86 |
| Warren | IN | 0.60 | 1 | 5 | 8.61 | 8413 | 1056 | 13.04 |
| Washington | MO | 1.00 | 1 | 5 | 4.90 | 23499 | 6664 | 28.13 |
| Wayne | IL | 0.00 | 10 | 33 | 3.18 | 16121 | 2720 | 16.75 |
| Wayne | TN | 0.00 | 11 | 33 | 3.03 | 16202 | 9071 | 29.32 |
