# Supplementary material for: Concurrent disease burden from multiple infectious diseases and the influence of social determinants in the contiguous United States
Source: PLoS One. 2024 Sep 4;19(9):e0293431. doi: 10.1371/journal.pone.0293431 (PMC11373817; doi:10.1371/journal.pone.0293431)
Supplement: S7 File — Included in the table are the county name, state, p-value, expected number of cases, observed number of cases, the relative risk for the disease, the county population, the number of individuals below the poverty line in the county, and the percent of the county population that is 125% below the US poverty line. (DOCX) [file pone.0293431.s007.docx]

**Supporting Information**

**S7 File**

**Tables Y-AB.** The following tables compare the counties that had a high relative risk for the same disease in consecutive years, adjusted for the percent of the county that was 125% below the poverty level. Included in the table are the county name, state, p-value, expected number of cases, observed number of cases, the relative risk for the disease, the county population, the number of individuals below the poverty line in the county, and the percent of the county population that is 125% below the US poverty line.

**Table Y. COVID-19 2021 & 2022.**

| *Disease* | *County* | *State* | *P-Value* | *Expected* | *Observed* | *Relative Risk* | *Population* | *POV* | *125percbelow* |
| --- | --- | --- | --- | --- | --- | --- | --- | --- | --- |
| COVID-19 2021 | Benton | TN | 0.00 | 174 | 753 | 4.32 | 15812 | 3973 | 24.86 |
| COVID-19 2022 | Benton | TN | 0.00 | 9890 | 46855 | 4.74 | 15812 | 3973 | 24.86 |
| COVID-19 2021 | Clearwater | MN | 0.00 | 567 | 1224 | 2.16 | 13954 | 1736 | 19.94 |
| COVID-19 2022 | Clearwater | MN | 0.00 | 1880 | 2920 | 1.55 | 13954 | 1736 | 19.94 |
| COVID-19 2021 | Concho | TX | 0.00 | 122 | 210 | 1.72 | 3319 | 438 | 21.32 |
| COVID-19 2022 | Concho | TX | 0.00 | 512 | 793 | 1.55 | 3319 | 438 | 21.32 |
| COVID-19 2021 | Crowley | CO | 0.00 | 860 | 1660 | 1.93 | 5893 | 1375 | 30.21 |
| COVID-19 2022 | Crowley | CO | 0.00 | 1758 | 2499 | 1.42 | 5893 | 1375 | 30.21 |
| COVID-19 2021 | Effingham | GA | 0.00 | 1620 | 2722 | 1.68 | 65150 | 6975 | 11.32 |
| COVID-19 2022 | Effingham | GA | 0.00 | 5891 | 9250 | 1.57 | 65150 | 6975 | 11.32 |
| COVID-19 2021 | Erie | OH | 0.00 | 2735 | 4912 | 1.80 | 75429 | 11853 | 16.18 |
| COVID-19 2022 | Erie | OH | 0.00 | 72786 | 151262 | 2.08 | 75429 | 11853 | 16.18 |
| COVID-19 2021 | Gila | AZ | 0.00 | 2663 | 4552 | 1.71 | 53306 | 13883 | 26.28 |
| COVID-19 2022 | Gila | AZ | 0.00 | 8044 | 12274 | 1.53 | 53306 | 13883 | 26.28 |
| COVID-19 2021 | Hockley | TX | 0.00 | 1370 | 1906 | 1.39 | 21490 | 4565 | 20.60 |
| COVID-19 2022 | Hockley | TX | 0.00 | 3407 | 4668 | 1.37 | 21490 | 4565 | 20.60 |
| COVID-19 2021 | Jeff Davis | TX | 0.00 | 48 | 108 | 2.25 | 1978 | 7563 | 24.48 |
| COVID-19 2022 | Jeff Davis | TX | 0.00 | 1030 | 3028 | 2.94 | 1978 | 7563 | 24.48 |
| COVID-19 2021 | Kerr | TX | 0.00 | 1721 | 2380 | 1.38 | 52676 | 8864 | 17.50 |
| COVID-19 2022 | Kerr | TX | 0.00 | 5346 | 7358 | 1.38 | 52676 | 8864 | 17.50 |
| COVID-19 2021 | Lee | KY | 0.00 | 487 | 1043 | 2.14 | 7360 | 2807 | 42.39 |
| COVID-19 2022 | Lee | KY | 0.00 | 9657 | 26975 | 2.79 | 7360 | 2807 | 42.39 |
| COVID-19 2021 | Mississippi | MO | 0.00 | 263 | 1139 | 4.32 | 12537 | 4083 | 35.18 |
| COVID-19 2022 | Mississippi | MO | 0.00 | 1911 | 9527 | 4.99 | 12537 | 4083 | 35.18 |
| COVID-19 2021 | Montrose | CO | 0.00 | 1300 | 2134 | 1.64 | 42798 | 6851 | 16.37 |
| COVID-19 2022 | Montrose | CO | 0.00 | 4119 | 7030 | 1.71 | 42798 | 6851 | 16.37 |
| COVID-19 2021 | Murray | MN | 0.00 | 4104 | 5160 | 1.26 | 39756 | 925 | 11.45 |
| COVID-19 2022 | Murray | MN | 0.01 | 13028 | 13620 | 1.05 | 30351 | 925 | 11.45 |
| COVID-19 2021 | Nicollet | MN | 0.00 | 2309 | 3094 | 1.34 | 49292 | 3907 | 12.28 |
| COVID-19 2022 | Nicollet | MN | 0.00 | 7232 | 8525 | 1.18 | 49292 | 3907 | 12.28 |
| COVID-19 2021 | Ozaukee | WI | 0.00 | 5962 | 7115 | 1.19 | 91569 | 6664 | 7.65 |
| COVID-19 2022 | Ozaukee | WI | 0.00 | 14764 | 15937 | 1.08 | 91569 | 6664 | 7.65 |
| COVID-19 2021 | Pope | IL | 0.00 | 770 | 1089 | 1.41 | 17869 | 1065 | 26.50 |
| COVID-19 2022 | Pope | IL | 0.00 | 28389 | 49153 | 1.73 | 67289 | 1065 | 26.50 |
| COVID-19 2021 | Richland | IL | 0.00 | 602 | 875 | 1.45 | 15803 | 2397 | 15.56 |
| COVID-19 2022 | Richland | IL | 0.00 | 31467 | 34848 | 1.11 | 105832 | 2397 | 15.56 |
| COVID-19 2021 | Richmond | VA | 0.00 | 651 | 819 | 1.26 | 8920 | 1976 | 27.20 |
| COVID-19 2022 | Richmond | VA | 0.00 | 9032 | 38075 | 4.22 | 8920 | 1976 | 27.20 |
| COVID-19 2021 | Schoharie | NY | 0.65 | 484 | 572 | 1.18 | 29720 | 4685 | 15.70 |
| COVID-19 2022 | Schoharie | NY | 0.00 | 2986 | 3470 | 1.16 | 29720 | 4685 | 15.70 |
| COVID-19 2021 | Seminole | OK | 0.00 | 322 | 688 | 2.14 | 23508 | 7058 | 29.52 |
| COVID-19 2022 | Seminole | OK | 0.00 | 14007 | 71009 | 5.08 | 23508 | 7058 | 29.52 |
| COVID-19 2021 | Stanly | NC | 0.00 | 1774 | 4331 | 2.44 | 62609 | 10197 | 16.95 |
| COVID-19 2022 | Stanly | NC | 0.00 | 5069 | 13128 | 2.59 | 62609 | 10197 | 16.95 |
| COVID-19 2021 | Trempealeau | WI | 0.00 | 2521 | 3064 | 1.22 | 30776 | 3648 | 12.57 |
| COVID-19 2022 | Trempealeau | WI | 0.00 | 4929 | 6192 | 1.26 | 30776 | 3648 | 12.57 |
| COVID-19 2021 | Union | KY | 0.00 | 395 | 537 | 1.36 | 13646 | 2974 | 20.90 |
| COVID-19 2022 | Union | KY | 0.00 | 4549 | 6594 | 1.45 | 13646 | 2974 | 20.90 |

**Table Z. HIV 2019 & 2020.**

| *Disease* | *County* | *State* | *P-Value* | *Expected* | *Observed* | *Relative Risk* | *Population* | *POV* | *125percbelow* |
| --- | --- | --- | --- | --- | --- | --- | --- | --- | --- |
| HIV 2019 | Allen | LA | 0.01 | 119 | 176 | 1.48 | 22750 | 4210 | 20.16 |
| HIV 2020 | Allen | LA | 0.00 | 126 | 194 | 1.54 | 22750 | 4210 | 20.16 |
| HIV 2019 | Allendale | SC | 0.01 | 16 | 40 | 2.50 | 7990 | 2807 | 36.10 |
| HIV 2020 | Allendale | SC | 0.01 | 16 | 39 | 2.49 | 7990 | 2807 | 36.10 |
| HIV 2019 | Austin | TX | 0.01 | 28 | 59 | 2.10 | 30109 | 5403 | 18.26 |
| HIV 2020 | Austin | TX | 0.02 | 26 | 54 | 2.10 | 30109 | 5403 | 18.26 |
| HIV 2019 | Benton | TN | 0.66 | 1 | 7 | 6.20 | 15812 | 3973 | 24.86 |
| HIV 2020 | Benton | TN | 0.66 | 1 | 7 | 6.20 | 15812 | 3973 | 24.86 |
| HIV 2019 | Columbia | AR | 0.00 | 51 | 138 | 2.68 | 74020 | 6505 | 29.85 |
| HIV 2020 | Columbia | AR | 0.00 | 51 | 141 | 2.78 | 74020 | 6505 | 29.85 |
| HIV 2019 | Dillon | SC | 0.00 | 39 | 146 | 3.75 | 28284 | 10596 | 35.28 |
| HIV 2020 | Dillon | SC | 0.00 | 40 | 146 | 3.69 | 28284 | 10596 | 35.28 |
| HIV 2019 | Doddridge | WV | 0.60 | 1 | 8 | 5.48 | 7786 | 1611 | 20.97 |
| HIV 2020 | Doddridge | WV | 0.59 | 1 | 8 | 5.48 | 7786 | 1611 | 20.97 |
| HIV 2019 | Dooly | GA | 0.01 | 30 | 61 | 2.02 | 11142 | 3133 | 25.88 |
| HIV 2020 | Dooly | GA | 0.00 | 25 | 55 | 2.18 | 11142 | 3133 | 25.88 |
| HIV 2019 | Dorchester | MD | 0.00 | 78 | 148 | 1.91 | 32508 | 6543 | 20.75 |
| HIV 2020 | Dorchester | MD | 0.00 | 78 | 147 | 1.88 | 32508 | 6543 | 20.75 |
| HIV 2019 | Falls | TX | 0.00 | 18 | 45 | 2.46 | 16961 | 4790 | 30.84 |
| HIV 2020 | Falls | TX | 0.00 | 19 | 46 | 2.46 | 16961 | 4790 | 30.84 |
| HIV 2019 | Gila | AZ | 0.54 | 25 | 47 | 1.85 | 53306 | 13883 | 26.28 |
| HIV 2020 | Gila | AZ | 0.23 | 29 | 54 | 1.85 | 53306 | 13883 | 26.28 |
| HIV 2019 | Grady | GA | 0.25 | 13 | 30 | 2.37 | 26221 | 5712 | 23.43 |
| HIV 2020 | Grady | GA | 0.33 | 12 | 28 | 2.37 | 26221 | 5712 | 23.43 |
| HIV 2019 | Greensville | VA | 0.00 | 39 | 79 | 2.01 | 11393 | 1491 | 18.45 |
| HIV 2020 | Greensville | VA | 0.03 | 37 | 69 | 1.88 | 11393 | 1491 | 18.45 |
| HIV 2019 | Greer | OK | 0.02 | 8 | 26 | 3.06 | 5488 | 1235 | 25.93 |
| HIV 2020 | Greer | OK | 0.17 | 7 | 20 | 3.06 | 5488 | 1235 | 25.93 |
| HIV 2019 | Haywood | TN | 0.00 | 31 | 77 | 2.46 | 17795 | 4609 | 26.89 |
| HIV 2020 | Haywood | TN | 0.00 | 33 | 79 | 2.42 | 17795 | 4609 | 26.89 |
| HIV 2019 | Hood River | OR | 1.00 | 27 | 46 | 1.68 | 34102 | 2062 | 8.93 |
| HIV 2020 | Hood River | OR | 0.97 | 27 | 46 | 1.70 | 34102 | 2062 | 8.93 |
| HIV 2019 | Huerfano | CO | 0.65 | 3 | 12 | 3.69 | 6810 | 1230 | 18.70 |
| HIV 2020 | Huerfano | CO | 0.20 | 4 | 15 | 3.69 | 6810 | 1230 | 18.70 |
| HIV 2019 | Irwin | GA | 0.00 | 33 | 72 | 2.20 | 9643 | 2712 | 29.37 |
| HIV 2020 | Irwin | GA | 0.00 | 34 | 76 | 2.20 | 9643 | 2712 | 29.37 |
| HIV 2019 | Karnes | TX | 0.74 | 56 | 86 | 1.52 | 14721 | 2748 | 22.56 |
| HIV 2020 | Karnes | TX | 0.95 | 56 | 83 | 1.48 | 14721 | 2748 | 22.56 |
| HIV 2019 | Kerr | TX | 0.04 | 35 | 66 | 1.87 | 52676 | 8864 | 17.50 |
| HIV 2020 | Kerr | TX | 0.03 | 38 | 71 | 1.87 | 52676 | 8864 | 17.50 |
| HIV 2019 | Little River | AR | 0.08 | 8 | 23 | 3.03 | 27749 | 2296 | 19.00 |
| HIV 2020 | Little River | AR | 0.05 | 8 | 24 | 3.01 | 27749 | 2296 | 19.00 |
| HIV 2019 | Manistee | MI | 0.12 | 24 | 49 | 2.00 | 117876 | 3494 | 15.04 |
| HIV 2020 | Manistee | MI | 0.48 | 26 | 48 | 1.85 | 117876 | 3494 | 15.04 |
| HIV 2019 | Nacogdoches | TX | 0.00 | 60 | 108 | 1.80 | 64624 | 17878 | 30.04 |
| HIV 2020 | Nacogdoches | TX | 0.00 | 61 | 112 | 1.83 | 64624 | 17878 | 30.04 |
| HIV 2019 | Nottoway | VA | 0.03 | 70 | 113 | 1.61 | 15614 | 3114 | 22.96 |
| HIV 2020 | Nottoway | VA | 0.50 | 54 | 85 | 1.56 | 15614 | 3114 | 22.96 |
| HIV 2019 | Pacific | WA | 0.79 | 18 | 35 | 1.97 | 23470 | 4083 | 18.80 |
| HIV 2020 | Pacific | WA | 0.41 | 17 | 36 | 2.08 | 23470 | 4083 | 18.80 |
| HIV 2019 | Pontotoc | MS | 0.89 | 12 | 26 | 2.16 | 31206 | 7038 | 22.23 |
| HIV 2020 | Pontotoc | MS | 0.89 | 12 | 26 | 2.16 | 31206 | 7038 | 22.23 |
| HIV 2019 | Richmond | VA | 0.00 | 6 | 28 | 4.86 | 8920 | 1976 | 27.20 |
| HIV 2020 | Richmond | VA | 0.00 | 6 | 28 | 4.86 | 8920 | 1976 | 27.20 |
| HIV 2019 | San Juan | UT | 0.16 | 36 | 64 | 1.80 | 110842 | 4131 | 27.72 |
| HIV 2020 | San Juan | UT | 0.08 | 40 | 71 | 1.78 | 110842 | 4131 | 27.72 |
| HIV 2019 | Simpson | MS | 0.00 | 314 | 486 | 1.55 | 238704 | 6898 | 26.29 |
| HIV 2020 | Simpson | MS | 0.00 | 313 | 480 | 1.53 | 238704 | 6898 | 26.29 |
| HIV 2019 | Stanly | NC | 0.00 | 35 | 97 | 2.75 | 62609 | 10197 | 16.95 |
| HIV 2020 | Stanly | NC | 0.00 | 39 | 106 | 2.75 | 62609 | 10197 | 16.95 |
| HIV 2019 | Talbot | GA | 0.05 | 7 | 23 | 3.10 | 5731 | 1829 | 29.46 |
| HIV 2020 | Talbot | GA | 0.08 | 7 | 22 | 3.06 | 5731 | 1829 | 29.46 |
| HIV 2019 | Treutlen | GA | 0.00 | 37 | 96 | 2.60 | 37697 | 2330 | 36.40625 |
| HIV 2020 | Treutlen | GA | 0.00 | 33 | 86 | 2.61 | 37697 | 2330 | 36.40625 |
| HIV 2019 | Wilkinson | MS | 0.00 | 186 | 315 | 1.70 | 72637 | 2601 | 34.65 |
| HIV 2020 | Wilkinson | MS | 0.00 | 98 | 169 | 1.73 | 72637 | 2601 | 34.65 |
| HIV 2019 | Woodford | KY | 0.97 | 9 | 21 | 2.28 | 26892 | 3336 | 12.81 |
| HIV 2020 | Woodford | KY | 0.97 | 9 | 21 | 2.28 | 26892 | 3336 | 12.81 |

**Table AA. INFLUENZA 2020 & 2021.**

| *Disease* | *County* | *State* | *P-Value* | *Expected* | *Observed* | *Relative Risk* | *Population* | *POV* | *125percbelow* |
| --- | --- | --- | --- | --- | --- | --- | --- | --- | --- |
| INFLUENZA 2020 | Gordon | GA | 0.00 | 7329 | 17388 | 2.37 | 57696 | 11962 | 20.99 |
| INFLUENZA 2021 | Gordon | GA | 0.00 | 22470 | 39414 | 1.75 | 57696 | 11962 | 20.99 |

**Table AB. TB 2019 & 2020.**

| *Disease* | *County* | *State* | *P-Value* | *Expected* | *Observed* | *Relative Risk* | *Population* | *POV* | *125percbelow* |
| --- | --- | --- | --- | --- | --- | --- | --- | --- | --- |
| TB 2019 | Fulton | IL | 0.06 | 21 | 44 | 2.11 | 33440 | 5972 | 18.61 |
| TB 2020 | Fulton | IL | 0.64 | 13 | 28 | 2.11 | 33440 | 5972 | 18.61 |
| TB 2019 | Fulton | IN | 0.00 | 16 | 44 | 2.69 | 20434 | 3907 | 19.84 |
| TB 2020 | Fulton | IN | 0.04 | 10 | 28 | 2.69 | 20434 | 3907 | 19.84 |
| TB 2019 | Monroe | AR | 0.02 | 3 | 15 | 4.45 | 6765 | 1978 | 29.14 |
| TB 2020 | Monroe | AR | 0.78 | 2 | 8 | 4.45 | 6765 | 1978 | 29.14 |
| TB 2019 | Richmond | VA | 0.00 | 5 | 22 | 4.86 | 8920 | 1976 | 27.20 |
| TB 2020 | Richmond | VA | 0.00 | 3 | 16 | 4.86 | 8920 | 1976 | 27.20 |
| TB 2019 | Warren | IN | 0.01 | 1 | 9 | 8.61 | 8413 | 1056 | 13.04 |
| TB 2020 | Warren | IN | 0.60 | 1 | 5 | 8.61 | 8413 | 1056 | 13.04 |
