# Supplementary material for: Concurrent disease burden from multiple infectious diseases and the influence of social determinants in the contiguous United States
Source: PLoS One. 2024 Sep 4;19(9):e0293431. doi: 10.1371/journal.pone.0293431 (PMC11373817; doi:10.1371/journal.pone.0293431)
Supplement: S8 File — Included in the table are the county name, state, p-value, expected number of cases, observed number of cases, the relative risk for the disease, the county population, the number of individuals below the poverty line in the county, and the percent of the county population that is 125% below the US poverty line. (DOCX) [file pone.0293431.s008.docx]

**Supporting Information**

**S8 File**

**Tables AC-AF.** The following tables list the counties that had a high relative risk for two different diseases, adjusted for the percent of the county that was below the poverty level. Included in the table are the county name, state, p-value, expected number of cases, observed number of cases, the relative risk for the disease, the county population, the number of individuals below the poverty line in the county, and the percent of the county population that is 125% below the US poverty line.

**Table AC. COVID-19 & HIV.**

| *Disease* | *County* | *State* | *P-Value* | *Expected* | *Observed* | *Relative Risk* | *Population* | *POV* | *125percbelow* |
| --- | --- | --- | --- | --- | --- | --- | --- | --- | --- |
| HIV 2019 | Allen 5 | LA | 0.01 | 119 | 176 | 1.48 | 22750 | 4210 | 20.16 |
| HIV 2020 | Allen 5 | LA | 0.00 | 126 | 194 | 1.54 | 22750 | 4210 | 20.16 |
| COVID-19 2022 | Allen 5 | LA | 0.00 | 45464 | 75061 | 1.65 | 22750 | 4210 | 20.16 |
| HIV 2019 | Austin | TX | 0.01 | 28 | 59 | 2.10 | 30109 | 5403 | 18.26 |
| HIV 2020 | Austin | TX | 0.02 | 26 | 54 | 2.10 | 30109 | 5403 | 18.26 |
| COVID-19 2021 | Austin | TX | 0.00 | 759 | 1037 | 1.37 | 30109 | 5403 | 18.26 |
| HIV 2019 | Baldwin 2 | GA | 0.00 | 66 | 129 | 1.95 | 43773 | 11540 | 28.28 |
| COVID-19 2021 | Baldwin 2 | GA | 0.00 | 2010 | 3245 | 1.61 | 43773 | 11540 | 28.28 |
| HIV 2019 | Benton 8 | TN | 0.66 | 1 | 7 | 6.20 | 15812 | 3973 | 24.86 |
| HIV 2020 | Benton 8 | TN | 0.66 | 1 | 7 | 6.20 | 15812 | 3973 | 24.86 |
| COVID-19 2021 | Benton 8 | TN | 0.00 | 174 | 753 | 4.32 | 15812 | 3973 | 24.86 |
| COVID-19 2022 | Benton 8 | TN | 0.00 | 9890 | 46855 | 4.74 | 15812 | 3973 | 24.86 |
| HIV 2019 | Columbia 3 | AR | 0.00 | 51 | 138 | 2.68 | 74020 | 6505 | 29.85 |
| HIV 2020 | Columbia 3 | AR | 0.00 | 51 | 141 | 2.78 | 74020 | 6505 | 29.85 |
| COVID-19 2021 | Columbia 3 | AR | 0.00 | 1999 | 4102 | 2.05 | 74020 | 6505 | 29.85 |
| HIV 2020 | Crowley | CO | 0.98 | 11 | 23 | 2.17 | 5893 | 1375 | 30.21 |
| COVID-19 2021 | Crowley | CO | 0.00 | 860 | 1660 | 1.93 | 5893 | 1375 | 30.21 |
| COVID-19 2022 | Crowley | CO | 0.00 | 1758 | 2499 | 1.42 | 5893 | 1375 | 30.21 |
| HIV 2019 | Doddridge | WV | 0.60 | 1 | 8 | 5.48 | 7786 | 1611 | 20.97 |
| HIV 2020 | Doddridge | WV | 0.59 | 1 | 8 | 5.48 | 7786 | 1611 | 20.97 |
| COVID-19 2021 | Doddridge | WV | 0.00 | 142 | 257 | 1.81 | 7786 | 1611 | 20.97 |
| COVID-19 2021 | Erie | OH | 0.00 | 2735 | 4912 | 1.80 | 75429 | 11853 | 16.18 |
| COVID-19 2022 | Erie | OH | 0.00 | 72786 | 151262 | 2.08 | 75429 | 11853 | 16.18 |
| HIV 2020 | Erie | OH | 0.54 | 59 | 91 | 1.53 | 75429 | 11853 | 16.18 |
| COVID-19 2021 | Falls | TX | 0.00 | 706 | 1199 | 1.70 | 16961 | 4790 | 30.84 |
| HIV 2019 | Falls | TX | 0.00 | 18 | 45 | 2.46 | 16961 | 4790 | 30.84 |
| HIV 2020 | Falls | TX | 0.00 | 19 | 46 | 2.46 | 16961 | 4790 | 30.84 |
| HIV 2019 | Gila | AZ | 0.54 | 25 | 47 | 1.85 | 53306 | 13883 | 26.28 |
| HIV 2020 | Gila | AZ | 0.23 | 29 | 54 | 1.85 | 53306 | 13883 | 26.28 |
| COVID-19 2021 | Gila | AZ | 0.00 | 2663 | 4552 | 1.71 | 53306 | 13883 | 26.28 |
| COVID-19 2022 | Gila | AZ | 0.00 | 8044 | 12274 | 1.53 | 53306 | 13883 | 26.28 |
| HIV 2019 | Grady | GA | 0.25 | 13 | 30 | 2.37 | 26221 | 5712 | 23.43 |
| HIV 2020 | Grady | GA | 0.33 | 12 | 28 | 2.37 | 26221 | 5712 | 23.43 |
| COVID-19 2021 | Grady | GA | 0.00 | 726 | 1341 | 1.85 | 26221 | 5712 | 23.43 |
| HIV 2019 | Greensville | VA | 0.00 | 39 | 79 | 2.01 | 11393 | 1491 | 18.45 |
| HIV 2020 | Greensville | VA | 0.03 | 37 | 69 | 1.88 | 11393 | 1491 | 18.45 |
| COVID-19 2021 | Greensville | VA | 0.00 | 516 | 1019 | 1.98 | 11393 | 1491 | 18.45 |
| HIV 2019 | Greer | OK | 0.02 | 8 | 26 | 3.06 | 5488 | 1235 | 25.93 |
| HIV 2020 | Greer | OK | 0.17 | 7 | 20 | 3.06 | 5488 | 1235 | 25.93 |
| COVID-19 2021 | Greer | OK | 0.00 | 326 | 580 | 1.78 | 7953 | 1235 | 25.93 |
| HIV 2020 | Hancock | GA | 0.00 | 76 | 142 | 1.88 | 59257 | 1806 | 29.62 |
| COVID-19 2022 | Hancock | GA | 0.00 | 30507 | 55255 | 1.81 | 81397 | 1806 | 29.62 |
| HIV 2019 | Hood River | OR | 1.00 | 27 | 46 | 1.68 | 34102 | 2062 | 8.93 |
| HIV 2020 | Hood River | OR | 0.97 | 27 | 46 | 1.70 | 34102 | 2062 | 8.93 |
| COVID-19 2021 | Hood River | OR | 0.00 | 1142 | 1744 | 1.53 | 34102 | 2062 | 8.93 |
| HIV 2019 | Irwin | GA | 0.00 | 33 | 72 | 2.20 | 9643 | 2712 | 29.37 |
| HIV 2020 | Irwin | GA | 0.00 | 34 | 76 | 2.20 | 9643 | 2712 | 29.37 |
| COVID-19 2021 | Irwin | GA | 0.00 | 321 | 674 | 2.10 | 9643 | 2712 | 29.37 |
| HIV 2019 | Kerr | TX | 0.04 | 35 | 66 | 1.87 | 52676 | 8864 | 17.50 |
| HIV 2020 | Kerr | TX | 0.03 | 38 | 71 | 1.87 | 52676 | 8864 | 17.50 |
| COVID-19 2021 | Kerr | TX | 0.00 | 1721 | 2380 | 1.38 | 52676 | 8864 | 17.50 |
| COVID-19 2022 | Kerr | TX | 0.00 | 5346 | 7358 | 1.38 | 52676 | 8864 | 17.50 |
| HIV 2019 | Manistee | MI | 0.12 | 24 | 49 | 2.00 | 117876 | 3494 | 15.04 |
| HIV 2020 | Manistee | MI | 0.48 | 26 | 48 | 1.85 | 117876 | 3494 | 15.04 |
| COVID-19 2021 | Manistee | MI | 0.00 | 419 | 609 | 1.45 | 25063 | 3494 | 15.04 |
| HIV 2019 | Mississippi | MO | 0.00 | 15 | 63 | 4.25 | 79271 | 4083 | 35.18 |
| COVID-19 2021 | Mississippi | MO | 0.00 | 263 | 1139 | 4.32 | 12537 | 4083 | 35.18 |
| COVID-19 2022 | Mississippi | MO | 0.00 | 1911 | 9527 | 4.99 | 12537 | 4083 | 35.18 |
| HIV 2019 | Nacogdoches | TX | 0.00 | 60 | 108 | 1.80 | 64624 | 17878 | 30.04 |
| HIV 2020 | Nacogdoches | TX | 0.00 | 61 | 112 | 1.83 | 64624 | 17878 | 30.04 |
| COVID-19 2021 | Nacogdoches | TX | 0.00 | 1267 | 3029 | 2.39 | 64624 | 17878 | 30.04 |
| HIV 2019 | Nottoway | VA | 0.03 | 70 | 113 | 1.61 | 15614 | 3114 | 22.96 |
| HIV 2020 | Nottoway | VA | 0.50 | 54 | 85 | 1.56 | 15614 | 3114 | 22.96 |
| COVID-19 2021 | Nottoway | VA | 0.00 | 873 | 1176 | 1.35 | 15614 | 3114 | 22.96 |
| HIV 2019 | Pontotoc | MS | 0.89 | 12 | 26 | 2.16 | 31206 | 7038 | 22.23 |
| HIV 2020 | Pontotoc | MS | 0.89 | 12 | 26 | 2.16 | 31206 | 7038 | 22.23 |
| COVID-19 2021 | Pontotoc | MS | 0.00 | 1520 | 3135 | 2.06 | 31206 | 7038 | 22.23 |
| HIV 2019 | Simpson | MS | 0.00 | 314 | 486 | 1.55 | 238704 | 6898 | 26.29 |
| HIV 2020 | Simpson | MS | 0.00 | 313 | 480 | 1.53 | 238704 | 6898 | 26.29 |
| COVID-19 2021 | Simpson | MS | 0.00 | 10424 | 14827 | 1.42 | 238704 | 6898 | 26.29 |
| HIV 2019 | Stanly | NC | 0.00 | 35 | 97 | 2.75 | 62609 | 10197 | 16.95 |
| HIV 2020 | Stanly | NC | 0.00 | 39 | 106 | 2.75 | 62609 | 10197 | 16.95 |
| COVID-19 2021 | Stanly | NC | 0.00 | 1774 | 4331 | 2.44 | 62609 | 10197 | 16.95 |
| COVID-19 2022 | Stanly | NC | 0.00 | 5069 | 13128 | 2.59 | 62609 | 10197 | 16.95 |
| HIV 2019 | Talbot | GA | 0.05 | 7 | 23 | 3.10 | 5731 | 1829 | 29.46 |
| HIV 2020 | Talbot | GA | 0.08 | 7 | 22 | 3.06 | 5731 | 1829 | 29.46 |
| COVID-19 2021 | Talbot | GA | 0.00 | 388 | 1185 | 3.05 | 5731 | 1829 | 29.46 |
| HIV 2019 | Treutlen | GA | 0.00 | 37 | 96 | 2.60 | 37697 | 2330 | 36.41 |
| HIV 2020 | Treutlen | GA | 0.00 | 33 | 86 | 2.61 | 37697 | 2330 | 36.41 |
| COVID-19 2021 | Treutlen | GA | 0.00 | 2466 | 4707 | 1.91 | 37697 | 2330 | 36.41 |
| HIV 2019 | Woodford | KY | 0.97 | 9 | 21 | 2.28 | 26892 | 3336 | 12.81 |
| HIV 2020 | Woodford | KY | 0.97 | 9 | 21 | 2.28 | 26892 | 3336 | 12.81 |
| COVID-19 2021 | Woodford | KY | 0.00 | 690 | 1253 | 1.82 | 26892 | 3336 | 12.81 |

**Table AD. COVID-19 & INFLUENZA.**

| *Disease* | *County* | *State* | *P-Value* | *Expected* | *Observed* | *Relative Risk* | *Population* | *POV* | *125percbelow* |
| --- | --- | --- | --- | --- | --- | --- | --- | --- | --- |
| COVID-19 2021 | Boyle | KY | 0.00 | 1203 | 2162 | 1.80 | 30701 | 5345 | 19.82 |
| INFLUENZA 2021 | Boyle | KY | 0.00 | 3677 | 6012 | 1.64 | 30701 | 5345 | 19.82 |
| COVID-19 2021 | Gordon | GA | 0.00 | 4265 | 4962 | 1.16 | 57696 | 11962 | 20.99 |
| INFLUENZA 2020 | Gordon | GA | 0.00 | 7329 | 17388 | 2.37 | 57696 | 11962 | 20.99 |
| INFLUENZA 2021 | Gordon | GA | 0.00 | 22470 | 39414 | 1.75 | 57696 | 11962 | 20.99 |

**Table AE. COVID-19 & TB.**

| *Disease* | *County* | *State* | *P-Value* | *Expected* | *Observed* | *Relative Risk* | *Population* | *POV* | *125percbelow* |
| --- | --- | --- | --- | --- | --- | --- | --- | --- | --- |
| COVID-19 2022 | Jefferson | IN | 0.00 | 68318 | 126583 | 1.85 | 33075 | 5403 | 18.18 |
| TB 2019 | Jefferson | IN | 0.97 | 13 | 25 | 2.00 | 93436 | 5403 | 18.18 |
| COVID-19 2022 | Jefferson | MS | 0.00 | 53589 | 126583 | 2.37 | 7254 | 2309 | 34.99 |
| TB 2019 | Jefferson | MS | 0.94 | 8 | 18 | 2.37 | 7254 | 2309 | 34.99 |
| COVID-19 2021 | Montgomery | AR | 0.00 | 1322 | 2316 | 1.75 | 8459 | 2138 | 24.26 |
| TB 2020 | Montgomery | AR | 1.00 | 6 | 14 | 2.44 | 147779 | 2138 | 24.26 |
| COVID-19 2021 | Seminole | OK | 0.00 | 322 | 688 | 2.14 | 23508 | 7058 | 29.52 |
| COVID-19 2022 | Seminole | OK | 0.00 | 14007 | 71009 | 5.08 | 23508 | 7058 | 29.52 |
| TB 2020 | Seminole | OK | 0.31 | 1 | 7 | 6.63 | 23508 | 7058 | 29.52 |
| COVID-19 2022 | Shelby | IA | 0.00 | 24371 | 41228 | 1.69 | 11699 | 1457 | 12.86 |
| TB 2020 | Shelby | IA | 1.00 | 21 | 36 | 1.71 | 11699 | 1457 | 12.86 |

**Table AF. COVID-19, HIV & TB.**

| *Disease* | *County* | *State* | *P-Value* | *Expected* | *Observed* | *Relative Risk* | *Population* | *POV* | *125percbelow* |
| --- | --- | --- | --- | --- | --- | --- | --- | --- | --- |
| COVID-19 2021 | Richmond | VA | 0.00 | 651 | 819 | 1.26 | 8920 | 1976 | 27.20 |
| COVID-19 2022 | Richmond | VA | 0.00 | 9032 | 38075 | 4.22 | 8920 | 1976 | 27.20 |
| HIV 2019 | Richmond | VA | 0.00 | 6 | 28 | 4.86 | 8920 | 1976 | 27.20 |
| HIV 2020 | Richmond | VA | 0.00 | 6 | 28 | 4.86 | 8920 | 1976 | 27.20 |
| TB 2019 | Richmond | VA | 0.00 | 5 | 22 | 4.86 | 8920 | 1976 | 27.20 |
| TB 2020 | Richmond | VA | 0.00 | 3 | 16 | 4.86 | 8920 | 1976 | 27.20 |
