# Supplementary material for: Concurrent disease burden from multiple infectious diseases and the influence of social determinants in the contiguous United States
Source: PLoS One. 2024 Sep 4;19(9):e0293431. doi: 10.1371/journal.pone.0293431 (PMC11373817; doi:10.1371/journal.pone.0293431)
Supplement: Attachment — Submitted filename: Comments.docx [file pone.0293431.s009.docx]

Abstract:

Please write numbers less than 10 in letters (in the whole of the text)

Introduction:

Please replace "vaccination rates" with vaccination coverage.

Please justify the necessity of this study and write the aim of the study, in the last paragraph of the introduction.

Methods:

What is your study design?

What are the existing data sets? Do you mean the surveillance system?

Please refer to the source of data. In addition, please provide the definition of TB, HIV, COVID-19, and influenza in the US surveillance system.

Please provide the definition of a cluster for TB, HIV, COVID-19, and Influenza.

How to determine the expected cases?

What is the definition of high-risk and low-risk areas and clusters?

Please define the dependent variable. How to measure the dependent variable?

What are the covariates, and how to measure them?

How to assess the socioeconomic status of counties?

Results

In the first paragraph of the results, describe your data. For example, number of counties and incidence of the mentioned diseases in each county, and percent of the population below the poverty line in each county.

The authors declared "The percent of the county population at or below 125% of the US poverty level in counties identified as part of clusters ranged from 7.65% to 44.8%" 125% is correct?
